# Supplementary material for: Seroprevalence of anti-Lassa Virus IgG antibodies in three districts of Sierra Leone: A cross-sectional, population-based study
Source: PLoS Negl Trop Dis. 2023 Feb 9;17(2):e0010938. doi: 10.1371/journal.pntd.0010938 (PMC9946222; doi:10.1371/journal.pntd.0010938)
Supplement: S1 Text — Table A in S1 Text. Selected communities’ locations, LASV IgG seroprevalence, and sizes. Table B in S1 Text. List of variables and their descriptions, answer choices, and analysis level collected from inception of the PEER Health study. Table C in S1 Text. LASV IgG Seroprevalence stratified by household, village, and chiefdom level, overall, and between all three Districts. Table D in S1 Text. Univariate analysis of factors associated with potential correlates of exposure to LASV quantified by IgG seropositivity. Table E in S1 Text. Stratified analysis of LASV IgG seropositivity potential correlates of exposure by geographic location, comparing Kenema District and Tonkolili District (reference group). Table F in S1 Text. Stratified analysis of LASV IgG seropositivity potential correlates of exposure by geographic location, comparing Kenema District and Port Loko District (reference group). Table G in S1 Text. Stratified analysis of LASV IgG seropositivity potential correlates of exposure by geographic location, comparing Tonkolili District and Port Loko District (reference group). Table H in S1 Text. Multivariate analysis to determine likelihood of household construction materials and fieldworker observations affecting levels of LASV IgG seroprevalence in a household. (DOCX) [file pntd.0010938.s001.docx]

**S1 Text**

**Supplemental Table A. Selected communities’ locations, seropositivity, and sizes.**

| **Village** | **GPS Coordinates** | **Community Size*** | **Participants** | **Seropositivity N (%)** |
| --- | --- | --- | --- | --- |
| **Kenema** | | | | |
| Bandawa | N 7.762661  W 11.36658 | Large | 156 | 8 (5.18) |
| Bendu | N 8.098046  W 10.970994 | Medium | 165 | 32 (19.39) |
| Benduma | N 8.231721  W 10.890665 | Medium | 156 | 30 (19.23) |
| Dodo | N 8.154537  W 11.17158 | Small | 184 | 8 (4.35) |
| Fala Wandor | N 8.282319  W 11.379355 | Small | 173 | 12 (6.94) |
| Foindu | N 8.104974  W 11.055531 | Large | 180 | 64 (35.56) |
| Gandahun | N 7.616507  W 11.446048 | Small | 203 | 43 (21.18) |
| Gawama | N 8.231582  W 11.343562 | Small | 170 | 4 (2.35) |
| Guaba | N 8.175211  W 11.20012 | Small | 96 | 78 (81.25) |
| Kokoru | N 7.630865  W 11.11798 | Medium | 142 | 0 (0.00) |
| Komendeh | N 8.061616  W 10.996173 | Small | 137 | 20 (14.60) |
| Komoru | N 7.995187  W 11.083379 | Small | 177 | 10 (5.65) |
| Konia | N 8.106163  W 11.010608 | Medium | 119 | 49 (41.18) |
| Kpalepalu | N 8.323698  W 11.370818 | Medium | 238 | 33 (13.87) |
| Kpallu | N 7.907588  W 11.085342 | Small | 223 | 1 (0.45) |
| Kpava | N 7.892324  W 11.131801 | Small | 119 | 2 (1.68) |
| Kpetema | N 8.274488  W 11.074731 | Medium | 203 | 169 (83.25) |
| Maleh | N 7.898208  W 11.046249 | Small | 240 | 16 (6.67) |
| Masihun | N 7.847216  W 11.117803 | Medium | 203 | 0 (0.00) |
| Massahun | N 8.051014  W 10.968487 | Small | 96 | 10 (10.42) |
| Ngeihun | N 8.174653  W 11.085466 | Medium | 239 | 84 (35.15) |
| Njorpowahun | N 7.744658  W 11.04871 | Small | 57 | 24 (42.11) |
| Patama | N 7.757858  W 11.078438 | Small | 234 | 19 (8.12) |
| Sandaru | N 7.769912  W 11.041639 | Medium | 132 | 6 (4.55) |
| Sawula | N 7.953218  W 11.31145 | Medium | 232 | 9 (3.88) |
| Sendimei | N 7.712857  W 11.388466 | Medium | 156 | 14 (8.97) |
| Talia | N 8.048327  W 11.033974 | Large | 176 | 62 (35.23) |
| Tangahun | N 8.186889  W 11.200752 | Small | 131 | 12 (9.16) |
| Tobanda | N 7.80875  W 11.368307 | Small | 215 | 110 (51.16) |
| Yumbuma | N 8.12103  W 11.009368 | Medium | 216 | 111 (51.39) |
| **Port Loko** | | | | |
| Conakry-Dee | N 8.698279  W 13.239524 | Small | 120 | 0 (0.00) |
| Gbaria-Mamaki | N 8.691447  W 12.692198 | Small | 84 | 9 (10.71) |
| Gbogbodo | N 8.725511  W 12.959058 | Small | 64 | 14 (21.88) |
| Hamdalai | N 8.760678  W 12.472747 | Small | 82 | 61 (74.39) |
| Kamasondo | N 8.870712  W 12.513812 | Medium | 132 | 40 (30.30) |
| Kofondu | N 8.896977  W 12.836979 | Small | 38 | 3 (7.89) |
| Mabureh-Buya | N 8.868382  W 12.477709 | Small | 61 | 3 (4.92) |
| Madina | N 8.900728  W 12.508055 | Small | 59 | 9 (15.25) |
| Mafundu | N 8.941122  W 12.857576 | Small | 114 | 36 (31.58) |
| Magbelai | N 8.470298  W 12.655005 | Small | 108 | 40 (37.04) |
| Magbontho | N 8.475743  W 12.648131 | Small | 84 | 0 (0.00) |
| Malal Rotain | N 8.907392  W 12.875606 | Small | 82 | 3 (3.66) |
| Mamankie | N 8.562514  W 13.130782 | Small | 81 | 0 (0.00) |
| Mamankie 2 | N 8.566092  W 13.143895 | Large | 156 | 0 (0.00) |
| Mana 2 | N 8.713723  W 12.969454 | Small | 95 | 9 (9.47) |
| Mange Bureh | N 8.920821  W 12.855978 | Large | 189 | 0 (0.00) |
| Mange Morie | N 8.922138  W 12.857411 | Small | 143 | 5 (3.50) |
| Mapawa | N 8.579579  W 13.143377 | Medium | 112 | 2 (1.79) |
| Maron | N 8.726211  W 12.687446 | Small | 34 | 14 (41.18) |
| Minthormore | N 9.014186  W 12.827678 | Small | 155 | 11 (7.10) |
| Patifu | N 8.791904  W 12.843518 | Small | 95 | 35 (36.84) |
| Robanka | N 8.731459  W 12.719125 | Small | 59 | 3 (5.08) |
| Ropolon | N 8.883433  W 12.493767 | Small | 60 | 6 (10.00) |
| Samkoya | N 8.7068  W 13.239553 | Small | 35 | 7 (20.00) |
| Yankoro | N 8.914929  W 12.856997 | Small | 36 | 12 (33.33) |
| **Tonkolili** | | | | |
| Goindema | N 8.584283  W 11.427915 | Small | 72 | 5 (6.94) |
| Kamankay | N 8.773874  W 11.852921 | Small | 98 | 6 (6.12) |
| Kangida | N 8.757046  W 11.966604 | Small | 71 | 22 (30.99) |
| Mabathof | N 8.643569  W 11.502716 | Small | 120 | 106 (88.33) |
| Mafanta | N 8.685366  W 11.931459 | Small | 129 | 13 (10.08) |
| Maforaka | N 8.63644  W 11.831444 | Small | 96 | 2 (2.08) |
| Magbonka | N 8.785286  W 11.871397 | Small | 65 | 6 (9.23) |
| Magbonko | N 8.860883  W 11.813135 | Small | 81 | 18 (22.22) |
| Makil-Bana | N 8.696487  W 11.921544 | Small | 113 | 15 (13.27) |
| Manenpeh | N 8.668915  W 11.922364 | Large | 168 | 2 (1.19) |
| Mange Bo Road | N 8.645331  W 11.84615 | Medium | 142 | 14 (9.86) |
| Maraka | N 8.757134  W 11.886451 | Small | 108 | 8 (7.41) |
| Masana | N 8.668719  W 11.883029 | Small | 59 | 8 (13.56) |
| Masonsingbi | N 8.635849  W 11.474979 | Medium | 120 | 22 (18.33) |
| Masorie | N 8.468829  W 12.205451 | Small | 108 | 7 (6.48) |
| Massanga | N 8.74794  W 11.839153 | Small | 131 | 6 (4.58) |
| Matainkay | N 8.714903  W 11.902135 | Large | 168 | 0 (0.00) |
| Matal | N 8.701285  W 11.938828 | Small | 100 | 0 (0.00) |
| Mathafulie | N 8.6353  W 11.902604 | Medium | 120 | 8 (6.67) |
| Mathan | N 8.767533  W 11.876654 | Medium | 119 | 1 (0.84) |
| Mathora | N 8.73444  W 11.903496 | Medium | 166 | 6 (3.61) |
| Mayan | N 8.658053  W 11.891744 | Small | 120 | 19 (15.83) |
| Robal | N 8.696331  W 11.921455 | Large | 167 | 4 (2.40) |
| Robonkay | N 8.634413  W 11.445015 | Small | 144 | 0 (0.00) |
| Rogbenthenel | N 8.741888  W 11.89697 | Small | 120 | 5 (4.17) |
| Romangoro | N 8.757152  W 11.886355 | Small | 120 | 36 (30.00) |
| Rowaka | N 8.631235  W 11.700727 | Small | 171 | 0 (0.00) |

*Community size was classified by the estimated population of each community. Small communities, n=65-375; medium communities, n=376-686; large communities, n=687-1000.

**Supplemental Table B. List of variables and their descriptions, answer choices, and analysis level collected from inception of the PEER Health study.**

| **Variable** | **Description** | **Level** |
| --- | --- | --- |
| Household number  (XXX; 001-999) | Household identification number; Randomly assigned | Household level |
| Identification number  (YY; 01-12) | Participant identification number; Randomly assigned after screening | Individual level |
| GPS Coordinates | Coordinates taken on GPS reader at the household’s front door  (N Latitude; W Longitude) | Household level |
| Village | Village of residence | Household level |
| Chiefdom | Chiefdom of residence | Household level |
| District | District of residence | Household level |
| Sex (M/F) | Sex of participant | Individual level |
| Age (in years) | Age of participant at time of enrollment | Individual level |
| Occupation  (Farming, Domestic, Trade, Education, Mining, Healthcare, Transportation, or Other) | Occupation of the participant at the time of enrollment (only applies to participants 15 years of age or older) | Individual level |
| IgG ELISA Result  (Seropositivity; positive or negative) | Result of ELISA, detecting human anti-LASV nucleoprotein (NP) immunoglobulin G (IgG), Lineage IV | Individual level |
| Household Seroprevalence | Percentage of LASV IgG positive within each household [(positive individuals/individuals sampled)*100] | Household level |
| Village seroprevalence | Percentage of LASV IgG positive individuals within each village | Village level |
| Chiefdom seroprevalence | Percentage of LASV IgG positive individuals within each chiefdom | Chiefdom level |
| District seroprevalence | Percentage of LASV IgG positive individuals within each district | District level |
| Community Size  Small – n=65-375  Medium – n=376-686  Large – n=687-1000 | Categorized size of each village | Household level |
| Proximity of household to bushes  (<5m, 5-20m, or >20m) | Measurement of distance of the household to wild land or bushland (measured by the fieldworker) | Household level |
| Proximity of household to vegetation  (<5m, 5-20m, or >20m) | Measurement of distance of the household to cultivated land (measured by the fieldworker) | Household level |
| Proximity of household to refuse  (<5m, 5-20m, or >20m) | Measurement of distance of the household to refuse or garbage pits (measured by the fieldworker) | Household level |
| Proximity of household to toilet  (<5m, 5-20m, or >20m) | Measurement of distance of the household to their used toilet facility (measured by the fieldworker) | Household level |
| Condition of toilet  (Very poor, poor, fair, good, or very good) | The categorized condition of the main toilet facility used by the household. | Household level |
| Proximity of household to water source  (<5m, 5-20m, or >20m) | Measurement of distance of the household to water source (measured by the fieldworker) | Household level |
| Type of water source  (hand pump well, stream, tap, open well, or other) | The type of water source used by the majority of the household. | Household level |
| Rodent holes  (Y/N) | Presence of rodent holes in household (externally or internally; assessed by fieldworker) | Household level |
| Roof material  (corrugated or thatched) | Materials used in the construction of the household’s roof | Household level |
| Wall material  (mud bricks, mud and sticks, or cement bricks) | Materials used in the construction of the household’s walls | Household level |
| Cement used  (Y/N) | Was cement used in the construction of the household? | Household level |
| Floor material  (mud only, mud with cement, cement without tile, cement with tile, or other) | Materials used in the construction of the household’s floor | Household level |
| Food storage – in the house (Y/N) | Is food stored inside of the household? | Household level |
| Food storage – in the room  (Y/N) | Is food stored inside the participant’s room or sleeping space? | Individual level |
| Water storage – in the house  (Y/N) | Is water stored inside of the household? | Household level |
| Water storage – in the room  (Y/N) | Is water stored inside the participant’s room or sleeping space? | Individual level |
| Water storage  (covered or uncovered) | If water is stored inside, is the water container covered? | Household level |
| Rodent feces – in the house  (Y/N) | Are there rodent feces present inside the house? | Household level |
| Rodent feces – in the room  (Y/N) | Are there rodent feces present inside the participant’s room or sleeping space? | Individual level |

**Supplemental Table C. Seroprevalence stratified by household, village, and chiefdom level, overall, and between all three Districts.**

| **Variable** | **Total**  N (%) | **Kenema**  N (%) | **Tonkolili**  N (%) | **Port Loko**  N (%) | **p-value*** |
| --- | --- | --- | --- | --- | --- |
| **Households N** | **903** | **433** | **272** | **198** |  |
| > 1 Seropositive resident | 324 (35.88) | 185 (42.73) | 70 (25.74) | 69 (34.85) | **<.001** |
| > 50% seropositivity | 209 (23.15) | 65 (15.01) | 14 (5.15) | 18 (9.09) | **<.001** |
| **Villages N** | **82** | **30** | **25** | **27** |  |
| 0-25% | 63 (73.86) | 20 (66.67) | 26 (86.67) | 19 (67.86) | .168 |
| 26-50% | 13 (19.32) | 6 (20.00) | 3 (10.00) | 8 (28.67) |  |
| > 50% | 6 (6.82) | 4 (13.33) | 1 (3.33) | 1 (3.57) |  |
| **Chiefdom N** | **26** | **10** | **6** | **10** |  |
| 0-10% | 12 (46.15) | 5 (50.00) | 4 (66.67) | 3 (30.00) | .232 |
| 11-20% | 6 (23.08) | 2 (20.00) | 0 (0.00) | 4 (40.00) |  |
| > 20% | 8 (30.77) | 3 (30.00) | 2 (33.33) | 3 (30.00) |  |

*Chi-square and Fisher’s exact test used for categorical variables. ANOVA used for comparison of mean values across districts.

**Supplemental Table D. Univariate analysis of factors associated with exposure to LASV quantified by IgG seropositivity.**

| **N=10642** | **OR (95% CI)** | **LCL** | **UCL** | **p-value*** |
| --- | --- | --- | --- | --- |
| **District** |  |  |  |  |
| Port Loko | 1.000 | - | - | - |
| Kenema | 2.211 | 1.466 | 3.335 | **<.001** |
| Tonkolili | 0.812 | .518 | 1.271 | .362 |
| **Age** (per 10-year increase) | 1.078 | 1.075 | 1.081 | **<.001** |
| **Sex** |  |  |  |  |
| Female | 0.853 | .844 | .862 | **<.001** |
| **Occupation**** |  |  |  |  |
| Domestic | 1.000 | - | - | - |
| Transportation | 4.038 | 3.730 | 4.372 | **<.001** |
| Healthcare | 1.598 | 1.501 | 1.702 | **<.001** |
| Other | 1.325 | 1.275 | 1.377 | **<.001** |
| Farming | 1.214 | 1.194 | 1.234 | **<.001** |
| Mining | 1.06 | 1.025 | 1.103 | **.001** |
| Education | 1.003 | .981 | 1.026 | .797 |
| Trade | 0.997 | .975 | 1.021 | .826 |

*Chi-square and Fisher’s exact test used for categorical variables. T-tests used for continuous variables.

**Occupation analyzed only for those participants over the age of 15 years (n = 6875).

**Supplemental Table E. Stratified analysis of LF IgG seropositivity exposure risks by geographic location, comparing Kenema District and Tonkolili District (reference group)**

| **N=8364** | **OR (95% CI)** | **LCL** | **UCL** | **p-value*** |
| --- | --- | --- | --- | --- |
| **Age** (per 10-year increase) | 2.672 | 1.884 | 3.789 | **<.001** |
| **Sex** |  |  |  |  |
| Female | 1.718 | 1.406 | 3.017 | **<.001** |
| Male | 3.867 | 2.738 | 5.462 | **<.001** |
| **Occupation**** |  |  |  |  |
| Education | 8.838 | 4.257 | 16.500 | **<.001** |
| Transportation | 5.636 | 1.344 | 23.631 | **<.001** |
| Trade | 5.325 | 1.344 | 23.631 | **.018** |
| Farming | 5.261 | 3.581 | 7.730 | **<.001** |
| Domestic | 2.428 | 1.582 | 3..725 | **<.001** |
| Healthcare | .668 | .075 | 5.928 | .718 |

*Chi-square and Fisher’s exact test used for categorical variables. T-tests used for continuous variables.

**Occupation analyzed only for those participants over the age of 15 years (n=5282).

***Mining and “Other” were unable to be calculated due to small frequency count.

**Supplemental Table F. Stratified analysis of LF IgG seropositivity exposure risks by geographic location, comparing Kenema District and Port Loko District (reference group)**

| **N=7446** | **OR (95% CI)** | **LCL** | **UCL** | **p-value*** |
| --- | --- | --- | --- | --- |
| **Age** (per 10-year increase) | 2.255 | 1.497 | 3.399 | **<.001** |
| **Sex** |  |  |  |  |
| Female | 1.823 | 1.168 | 2.856 | **.008** |
| Male | 2.735 | 1.847 | 4.050 | **<.001** |
| **Occupation**** |  |  |  |  |
| Education | 6.783 | 2.861 | 16.079 | **<.001** |
| Trade | 7.54 | 2.998 | 18.962 | **<.001** |
| Farming | 3.084 | 1.956 | 4.860 | **<.001** |
| Domestic | 1.573 | .963 | 2.569 | .071 |
| Healthcare | .199 | .018 | 2.261 | .019 |

*Chi-square and Fisher’s exact test used for categorical variables. T-tests used for continuous variables.

**Occupation analyzed only for those participants over the age of 15 years (n=4761).

***Transportation and “Other” were unable to be calculated due to small frequency count.

**Supplemental Table G. Stratified analysis of LF IgG seropositivity exposure risks by geographic location, comparing Tonkolili District and Port Loko District (reference group)**

| **N=5474** | **OR (95% CI)** | **LCL** | **UCL** | **p-value*** |
| --- | --- | --- | --- | --- |
| **Age** (per 10-year increase) | .841 | .510 | 1.387 | .229 |
| **Sex** |  |  |  |  |
| Female | 1.645 | .540 | 1.427 | .599 |
| Male | .709 | .412 | 1.218 | .213 |
| **Occupation**** |  |  |  |  |
| Trade | 1.05 | .452 | 2.442 | .909 |
| Education | .673 | .349 | 1.299 | .238 |
| Domestic | .644 | .369 | 1.126 | .123 |
| Farming | .570 | .311 | 1.045 | .069 |
| Healthcare | .297 | .021 | 4.200 | .369 |

*Chi-square and Fisher’s exact test used for categorical variables. T-tests used for continuous variables.

**Occupation analyzed only for those participants over the age of 15 years (n=3707).

***Mining, Transportation, and “Other” were unable to be calculated due to small frequency count.

**Supplemental Table H. Multivariate analysis to determine likelihood of household construction materials and fieldworker observations effecting levels of seroprevalence in the household.**

| **Variable** | **Low Seroprevalence (<25%; N(%))** | **Moderate Seroprevalence (25-50%; N(%))** | **High Seroprevalence (>50%; N(%))** | **p-value*** |
| --- | --- | --- | --- | --- |
| **Proximity to bushes** |  |  |  |  |
| <5m | 391 (53.86) | 33 (41.25) | 37 (47.44) | .058 |
| 5-20m | 252 (34.71) | 37 (46.25) | 36 (46.15) |  |
| >20m | 83 (11.43) | 10 (12.50) | 5 (6.41) |  |
| **Proximity to refuse** |  |  |  |  |
| <5m | 360 (49.59) | 31 (38.75) | 31 (39.74) | .056 |
| 5-20m | 267 (36.78) | 36 (45.00) | 40 (51.28) |  |
| >20m | 99 (13.64) | 13 (16.25) | 7 (8.97) |  |
| **Proximity to water source** |  |  |  |  |
| <5m | 217 (29.89) | 20 (25.00) | 27 (34.62) | **.001** |
| 5-20m | 316 (43.53) | 37 (46.25) | 45 (57.69) |  |
| >20m | 193 (26.58) | 23 (28.75) | 6 (7.69) |  |
| **Proximity to toilet** |  |  |  |  |
| <5m | 297 (40.91) | 27 (33.75) | 31 (39.74) | .525 |
| 5-20m | 301 (41.46) | 37 (46.25) | 37 (47.44) |  |
| >20m | 128 (17.63) | 16 (20.00) | 10 (12.82) |  |
| **Proximity to vegetation** |  |  |  |  |
| <5m | 351 (48.35) | 32 (40.00) | 37 (47.44) | .469 |
| 5-20m | 296 (40.77) | 36 (45.00) | 35 (44.87) |  |
| >20m | 79 (10.88) | 12 (15.00) | 6 (7.69) |  |
