# Supplementary figures and images for: Seroprevalence of anti-Lassa Virus IgG antibodies in three districts of Sierra Leone: A cross-sectional, population-based study
Source: PLoS Negl Trop Dis. 2023 Feb 9;17(2):e0010938. doi: 10.1371/journal.pntd.0010938 (PMC9946222; doi:10.1371/journal.pntd.0010938)

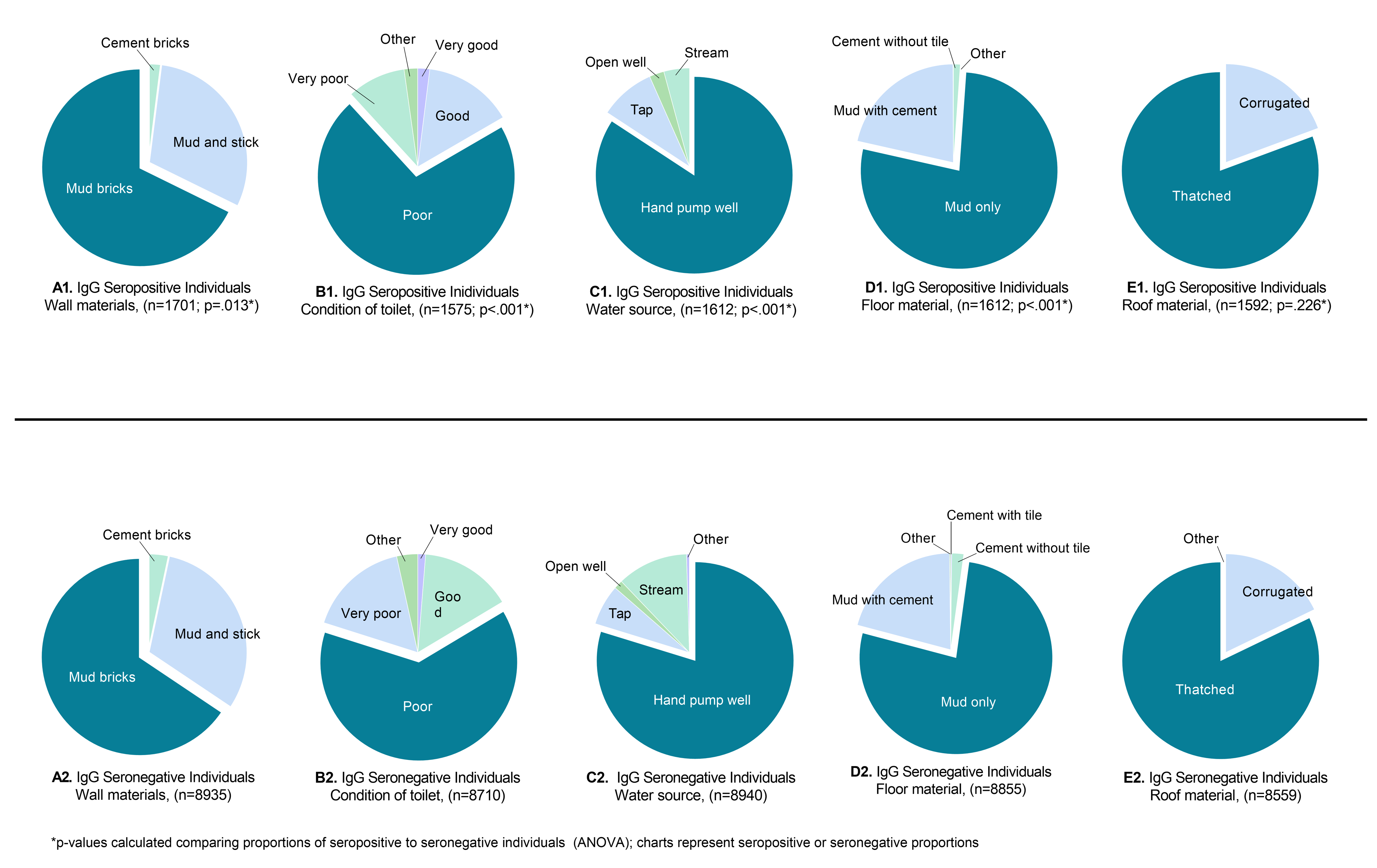

Supplement: S1 Fig — (A) Materials used in wall construction; (B) Fieldworker observation of the condition of household toilet facility; (C) Main type of water source used by household; (D) Materials used in floor construction; (E) Materials used in roof construction. (TIF) [file pntd.0010938.s002.tif]
