# Supplementary material for: Seroprevalence of anti-Lassa Virus IgG antibodies in three districts of Sierra Leone: A cross-sectional, population-based study
Source: PLoS Negl Trop Dis. 2023 Feb 9;17(2):e0010938. doi: 10.1371/journal.pntd.0010938 (PMC9946222; doi:10.1371/journal.pntd.0010938)
Supplement: Attachment — Submitted filename: PNTD-D-22-00744_responses_5Sep22.pdf [file pntd.0010938.s004.pdf]

Overall comments from PLOS NTD Editors: (please see all of our responses in blue)

Reviewers 1 and 2 both had some comments on limitations in the data, and how better to acknowledge them or account for them - please modify your discussion appropriately. Reviewer 1 and 2 had some difficulty understanding the statistical models and reporting used - be sure to explain it so that a non specialist reader can understand what was done and why. If any analyses need to be redone after assessing their comments please do so. Were the positive predictive value and sensitivity supposed to be the same number, or was this a typo? Reviewer 3 noted that some of the risk factors are difficult to address because of the retrospective nature of serology, be sure to acknowledge this in your discussion. Reviewer 2 noted that you had not provided precise information on the localities you worked - can this be provided as supplementary data?

We have significantly modified our discussion to include the comments about different limitations on the data recognized by reviewers 1 and 2. We have also changed and explained different statistical models and reports used to make sure that non-specialists will be able to understand our analysis more thoroughly. The data presented related to the LASV diagnostic kits was provided in the product insert and is beyond the scope of this study to provide anything outside of the manufacturer's instructions.

We have acknowledged the retrospective nature of the serology we used in our discussions and limitations at the end of our manuscript to address the concerns of Reviewer 3. We have also provided a supplementary table providing the precise information on the localities we worked with.

We would like to thank the reviewers for taking the time to read and critique our manuscript. Please find detailed responses to all critiques below.

Reviewer's Responses to Questions

Key Review Criteria Required for Acceptance?
As you describe the new analyses required for acceptance, please consider the following:

Methods
-Are the objectives of the study clearly articulated with a clear testable hypothesis stated?
-Is the study design appropriate to address the stated objectives?
-Is the population clearly described and appropriate for the hypothesis being tested?
-Is the sample size sufficient to ensure adequate power to address the hypothesis being tested?
-Were correct statistical analysis used to support conclusions?
-Are there concerns about ethical or regulatory requirements being met?

Reviewer #1: Methods
Line 152-156: this information is already presented in the introduction (see lines 116-124, which is the objective of the study.

These sentences have been removed from the methods section.

Line 157: "villages representative of small, medium, and large communities"; please give some clues about this categorization. What is small, the number of inhabitants, the surface? If the demography is the right parameter as I am thinking, so please give a number for each of them.

A more thorough description of small, medium, and large communities was added to the manuscript.

"After stratifying the districts by community size, villages representative of small (n=65-375), medium (n=376-686), and large (n=687-1000) communities were selected from each district. We hope this explains the categorization used during the selection of communities."

Are the small, medium and large communities represented equally in each district?

Villages were selected following the WHO cluster sampling method. This method has been used for many different cluster surveys in low- and middle-income countries since the 1970s and allows for weighted representation, to equally represent each district based on their population sizes.

Looking at your figure 1, we can easily estimate the number of individuals per village according to district; Kenema: 172 indiv/village (5162/30), Tonkolili: 118 indiv/village (3198/27) and Port Loko: 91 indiv/village (2278/25). Therefore, it seems that you have a decreasing gradient in sampling your villages from Kenema to Port Loko, showing that the sampling is not uniform, may be due to a higher proportion of large communities in Kenema?
May be due to a lower effort of sampling in the remote places from Kenema?

Please see the description above of our sampling method. Village selection was not done with simple random-sampling methods. All factors were taken into account to ensure the most statistically sound selection as possible.

Line 178-180: the precise location for each household was recorded using a GPS. It is therefore possible to give the coordinates of each village (see my main comment).

The reviewer is correct in that we collected GPS data for each household. However, since study inception, the investigators, in consultation with the Sierra Leone Ethics and Scientific Review Committee (SLESRC), decided that sharing household level GPS data could result in a loss of participant confidentiality. We are able to provide the names and central GPS coordinates of the villages, which chiefdoms they are in, and which districts they are in. Supplementary Table 1 has more precise information for each of the communities we visited.

Line 188-192: a table gathering all the variables would be very helpful.

We have provided more details about these variables in Supplementary Table 2.

Line 201-202: how do you define IgG prevalence? In other words what is the denominator: the individuals? The households?

Thank you for noting this need for clarification. We have explained the difference between seroprevalence and seropositivity in the text of the manuscript.

"IgG LASV seroprevalence was measured at the household level. IgG LASV seropositivity was measured at the individual level."

Line 214: add IgG between "LASV" and "prevalence"

Thank you for recognizing this omission. It has been added to the text.

"LASV IgG prevalence was determined from dried blood spots (DBS) by measuring the presence of IgG antibodies."

Line 222: you noted that "each replicate required 2-3 mm punches" per individual to run ELISA, but on the field (line 199), you noted that you collected enough blood to fill a single spot. Please harmonize the sampling between the field and the laboratory.

Thank you for pointing this out. In the field, it is correct that only a single blood spot was collected on a protein saver card. In the lab, this provides up to three 3 mm punches (or one 6mm punch) to be eluted. Then this processed sample can be run in duplicate on the ELISA per the manufacturer's instructions. We have edited the text to better clarify this method.

From the field – "Blood collection occurred in the subject's home. After performing a finger-stick with a lancet (1.5mm depth, 30g needle gauge BD Microtainer™ Contact-Activated Lancet, Becton, Dickinson and Company, Franklin Lakes, NJ), sufficient capillary blood was expressed to completely fill a single spot, which can provide up to three 3-mm punches to analyze, on Whatman™ 903 Protein Saver cards (Whatman Ltd., Piscataway, NJ)."

In the lab – "Each sample required two 3-mm punches, be eluted in 300uL (one 3-mm punch in 150uL per replicate) of sample buffer rocking at 4oC overnight."

Line 225-226: what is the difference between the sensitivity and positive predictive value, which have the same number? Same question between specificity and negative predictive value? I think this is a redundant information. Also, what is a diagnostic likelihood ratio? What are the comparable commercial diagnostics for Lassa, other pathogens?

We agree with the reviewer that positive and negative predictive value is redundant information and has been deleted from the text. We also removed the comparison to other commercial diagnostics because this point was not addressed in the current manuscript.

Reviewer #2: -Are the objectives of the study clearly articulated with a clear testable hypothesis stated? YES
-Is the study design appropriate to address the stated objectives? YES
-Is the population clearly described and appropriate for the hypothesis being tested? NO, the structure of the source population should be further described.

We have provided Supplementary Table 1 that describes the communities in more detail.

-Is the sample size sufficient to ensure adequate power to address the hypothesis being tested? YES, probably, but the question of sample size has not been addressed by authors in the manuscript.

Thank you for recognizing this omission. We have included Table 1 that describes the methods used to determine appropriate sample size.

-Were correct statistical analysis used to support conclusions? YES
-Are there concerns about ethical or regulatory requirements being met? NO

Reviewer #3: Nigeria, Sierra Leone and Liberia represent the epicentres of LASV epidemics. These regions record most epidemiological studies on LASV. While multiple crucial data on LASV fever are

needed (such as factors associated with high case fatality rate (approximately 30%), data on pregnant women, maternal-fetal outcomes, and strong evidence for new treatments and their integration in clinical care), there are already seroprevalence data demonstrating the endemicity of LASV in West Africa including Sierra Leone. Although the study by Grant et al. provides better information on the seroprevalence of LASV in Sierra Leone, it suffers from a lack of novelty and is limited to an extension of knowledge for new regions without being nationally representative of Sierra Leone.
The design of the study, which is also focused in past infections, further weakens the risk factors for acquiring LASV reported in the present study by the probable retrospective nature of the detection of IgG antibodies. Multiple categories of interest for LASV infections such as pregnant women are difficult to consider for these IgG detections because the infection cannot be confirmed as acquired before or after pregnancy.

We understand the reviewer's concerns about the novelty of this data. However, this is new data run on validated and confirmed LASV diagnostics kits. The last comprehensive field study of the epidemiology of Lassa in Sierra Leone was conducted in the 1980s, from McCormick et. al. This study gave a clearer picture of the presence of LASV throughout the country. Without neglecting the importance of the McCormick study, the Ministry of Health and Sanitation of Sierra Leone were prompted to investigate the potential for LASV to be present in newer geographic regions, particularly after 25-30 years. The goal of this study was not to provide a national representation of LASV in Sierra Leone, but to provide more information on the prevalence in districts with reportedly low levels of LASV seroprevalence.

We agree on the limitations of using IgG antibodies and the retrospective nature of serology. This will only represent the possibility of individuals being exposed to LASV at some point in the past, but there is no way for us to know when, where, why, or if they were symptomatic or asymptomatic. We decided to look at this serology and the risk factors of potential exposure, not necessarily risk factors for acquiring LASV. We hope the clarification made in the manuscript is sufficient to explain the reasons behind using serology.

We wholeheartedly agree about the importance of investigating other categories related to LASV risk factors and pathology, particularly in pregnant women, however, this was out of the scope of this study. We hope to expand on this knowledge in future epidemiological studies and research.

Results
-Does the analysis presented match the analysis plan?
-Are the results clearly and completely presented?
-Are the figures (Tables, Images) of sufficient quality for clarity?

Reviewer #1: Results
Line 238-: give the detailed information about the 82 villages in a table (see my comment above).

We have provided this information in Supplementary Table 1.

Line 261-271: This paragraph is very confusing because several ways of estimating proportions are presented in a descriptive way. The authors begin by using a median value that is not introduced in the M&M and does not reflect Figure 2A (line 261-263). Then they introduce the seroprevalence, which is assumed to be a percentage of the number of IgG positive individuals divided by the number

of individuals tested per village. Figures 2B-2D show the percentage of IgG positive households per village, which is not the same. This inflates the percentages and does not allow us to observe the variance in the distribution of IgG.

We apologize for this confusion. Reading back through this section we understand the confusion this explanation could cause. We have cleaned up this paragraph and added in more comments related to the methods. Earlier in the methods section, we add an explanation of our definitions of IgG seropositivity vs. IgG seroprevalence. IgG seropositivity will refer to individual level analyses, and IgG seroprevalence will refer to household level analyses. We hope this description and some rearranging of this paragraph helps clarify what we are trying to present.

Line 267-269: What is the point of presenting data by chiefdom if it is not supported by statistics?

We agree with this, so we have removed the description from the actual manuscript but kept the information in the supplemental table.

"The distribution of seroprevalence was visualized in ArcGIS at different levels within each district (Fig 2). When analyzed at the village level, the median seroprevalences of villages in Kenema, Port Loko, and Tonkolili Districts were 9.8% (IQR 4.7-35.2%), 9.7% (IQR 3.5-28.3%), and 6.6% (IQR 2.2-12.5%) respectively (Fig 2A). Kenema District had 10 villages with greater than 20% seroprevalence, which was higher than both Port Loko and Tonkolili Districts (33.0% (n=30), Fig 2B). There were eight villages in Port Loko District (32.0% (n = 25), Fig 2C) with greater than 20% seroprevalence, which was higher than Tonkolili District, with only four villages (14.8% (n = 27), Fig 2D), though this difference was not statistically significant. In Kenema District, 42.7% (n = 433; S3 Table) of households had one or more seropositive resident, Port Loko District had 37.1% (n = 198; S3 Table) households with one or more seropositive resident, and Tonkolili District had 23.8% (n = 272) (p<.001; S3 Table)."

Line 270-271: the percentages noted in the text for Tonkolili and Port Loko are different from those in table S1. Furthermore, the numbers of households written in the text are different from those in figure 1; Tonkolili: 272 in figure 1 vs 265 in the text, Port Loko: 198 in figure 1 vs 205 in the text. Please harmonize.

This was an error on our part. We have adjusted the text to harmonize the numbers across all text, figures, and tables.

Line 280-330: it seems that the authors are analyzing the data on positive individuals only.

This was another error. We have adjusted the explanation for the analysis of this data. We hope this continues to clarify our analyses and results. We have included a sample size in Figure 3 and hope this will help readers recognize how the analysis was conducted.

Line 283: the figure 4 is not good because each age class interval is different; 1 year (maybe a few months) for <1 year, 3 years for the 1-4 years interval, 10 years for the 5-14 years interval, 30 years for the 15-44 years interval and unknown for the over 45 years. This figure gives the impression that IgG seroprevalence is very high in the 15-44 age group, but in fact it is disproportionately high compared to the others because it includes the majority of individuals. Equivalent ranges and numbers of individuals per class should be presented in this figure. This would show why the test is not significant (line 283-284).

We had included these original age class intervals to match the original PEER proposal. However, reflecting on this analysis, we understand the confusion and how the figure could give the impression that IgG seroprevalence is disproportionately high compared to other groups. We have adjusted the figure to use a more uniform interval (that we had already run analysis on) and have adjusted Figure 4 to reflect this.

Line 320: p=0.013 is noted twice.

We have removed this extra p value from the manuscript.

Figures: they are all with a very low resolution. Please provide good ones.

We apologize for the poor resolution. We have provided better figures, and hopefully this will improve the readability of the figures.

Reviewer #2: -Does the analysis presented match the analysis plan? YES
-Are the results clearly and completely presented? To be improved (cf. comments)
-Are the figures (Tables, Images) of sufficient quality for clarity? NO, unsufficient resolution.

We have provided figures with clearer resolution and hope this improves the figures' appearances.

Reviewer #3: (No Response)

Conclusions
-Are the conclusions supported by the data presented?
-Are the limitations of analysis clearly described?
-Do the authors discuss how these data can be helpful to advance our understanding of the topic under study?
-Is public health relevance addressed?

Reviewer #1: Discussion
The discussion should include subheadings highlighting the variables of interest.

We have added in important subheadings to help better delineate which variables of interest are being discussed.

I am surprised to see no comparison with the data of McCormick et al. who had already shown a wide distribution of IgG in the country in the 1980s. Indeed, some communities were already infected between 10 and 52% in the eastern province, and between 10 and 15% in the northern province. As your data are not clearly presented, this is of course difficult to compare, especially for the eastern province (= Kenema district). According to your fig 2A, it seems that your IgG seroprevalences are similar to those observed 30 years ago by the McCormick team. This should be said somewhere.

This was an omission on our part. We have included a comparison of the range of seroprevalence from the McCormick team and our study. Our range was significantly wider than the McCormick study presented, which could further support our findings of increased LASV in Tonkolili and Port Loko Districts.

“Of particular interest is the difference between the northern province in the 1987 study compared to Tonkolili District, which is part of the northern province, in this study. McCormick et al. presented a range of 10-15% LASV seroprevalence.”

Line 362-366: this is very speculative. As you have the raw data, this can be verified easily.

While we agree, we unfortunately do not have similar enough raw data to the reference used. The reference used here looked at the proportion of LASV circulation among rodent-hosts, and not humans. We have clarified this in the text.

Line 427-429: what do you mean by validating a case-investigation form?

This is a reference to updated procedures at Kenema Government Hospital. We improved our methods of qualitative, quantitative, and mixed-methods surveys and questionnaires and have a process to formally validate any survey, form, or questionnaire. This helps standardize the data as well as the implementation and administration of the surveys, forms, or questionnaires. During this particular study, we did not have these procedures in place.

Reviewer #2: -Are the conclusions supported by the data presented? NO, not always (cf. comments).

These comments have been addressed in the attached document sent by the editors.

-Are the limitations of analysis clearly described? NO, unsufficiently (cf. comments).

These comments have also been addressed in the attached document.

-Do the authors discuss how these data can be helpful to advance our understanding of the topic under study? YES, but in a general way that is quite frustrating.

These comments have also been addressed in the attached document.

-Is public health relevance addressed? YES

Reviewer #3: (No Response)

Editorial and Data Presentation Modifications?
Use this section for editorial suggestions as well as relatively minor modifications of existing data that would enhance clarity. If the only modifications needed are minor and/or editorial, you may wish to recommend “Minor Revision” or “Accept”.

Reviewer #1: (No Response)

Reviewer #2: Cf. attached comments.

These comments have been addressed in the attached document sent by the editors.

Reviewer #3: (No Response)

Summary and General Comments
Use this section to provide overall comments, discuss strengths/weaknesses of the study, novelty, significance, general execution and scholarship. You may also include additional comments for the author, including concerns about dual publication, research ethics, or publication ethics. If requesting major revision, please articulate the new experiments that are needed.

Reviewer #1: This seroprevalence study of Lassa virus in Sierra Leone was eagerly awaited because the last data were collected in 1980-1985 (Mc Cormick et al. 1987). The authors made a remarkable investigation of 10,642 subjects, distributed in 3 districts. This is twice the sample analyzed by Mc Cormick (n = 5,213) in Sierra Leone and three times the sample analyzed by Lukashevich et al. in 1992 (3,126) in Guinea. Globally, the literature is extensive, accurate and updated.

However, the authors do not mention precisely the localities in which they worked, unlike their predecessors who reported the exact number of people tested per village, while naming the villages. This is one of my major criticism: the authors should present the data in a detailed table including: the district, the chiefdom, the village or locality with their geographical coordinates, the community size and the number of sera positive/tested sera. Indeed, the rough maps showing these locations in Figure 2 are clearly insufficient. Since each household has been geographically located with a Garmin GPS, this should not be a problem.

We appreciate these comments. We have provided a detailed supplementary table (Supplementary Table 1) that describes the localities more specifically. We have expanded on this in the comments above.

The variable "community size" should appear because it is an important epidemiological criterion. Indeed, we can think that in large communities, people are less close to the bushes or fields, which seems to be a risk factor in this study.

We have clarified the variable "community size" in a more quantitative way. The description can be found in the manuscript and in Supplementary Table 1.

"After stratifying the districts by community size, villages representative of small (n=65-375), medium (n=376-686), and large (n=687-1000) communities were selected from each district. We hope this explains the categorization used during the selection of communities."

There is also a big issue with the data analysis:
- a descriptive analysis taking into account all the results (positive and negative)
- a statistical analysis (multivariate) taking into account the positive results only.
Why doesn't the multivariate analysis take into account all the data (positive + negative)? This would allow for a robust analysis by taking into account the variance of each parameter. Because presented this way, one wonders why the variables are analyzed several times? For example, the "district" variable was analyzed twice: line 260-271, then line 289-303.

It also seems that the multivariate analysis in the results (line 280-307) presents the data by individual, while the second part (line 308-330) presents the data by household. At the end, we don't

know which parameters are relevant and which are really significant because of the change of outcome variable between one analysis and the other!

We apologize again for the confusion of this data analysis. The way we previously presented the data in the figures and tables was confusing and we understand how it might have looked as though we were only taking the positive results into account. We have adjusted the figures and removed a table to help the reader better understand the analysis of our variables, and how we included all data (positive and negative) for all variables of interest.

Abstract

Line 30: the keywords do not describe well your study. Pease add some others, such as IgG, prevalence, housing quality, etc

We have added in some additional keywords to more accurately describe our study.

"IgG, Housing Quality, Seroprevalence"

Introduction

Line 83: "the multimammate rat" should be "the Natal multimammate mouse"

We have corrected this misnomer in the text.

Line 89: why do you write LF and LASV infection rates? I think this is LASV infection only.

Thank you for noting this mistake. It has been edited in the manuscript.

Line 90-92: please update the numbers with more recent studies.

Thank you for the reminder. We have updated the numbers accordingly.

Line 124-130: these 4 sentences present the main findings of the study, as it is fine in a summary. This is inaccurate at the end of the introduction. Please change this part.

This was a good catch! We have moved these sentences to the discussion.

Reviewer #2: Cf. attached comments.

These comments have been addressed in the attached document sent by the editors.

Reviewer #3: (No Response)

Reviewer 2's attached document (provided by the editors):

These comments from reviewer 2 were attached in a separate document. We have copied and responded to these comments in one large document for ease of response.

General comments

This is a large sampled and well-conducted population-based study of LASV IgG seroprevalence in three district of Sierra Leone. Such a large scaled seroprevalence study have not been conducted in this part of West Africa since the 1980s. Moreover, this is the first time that improved serodiagnotics are used to conduct a large LASV seroprevalence study.

Overall, the methods used are accurate, except the calculation of sample size which is absent from the manuscript. I am less convinced by the interpretation of some of the results made by the authors. Some affirmations are not supported by the results or by the cited references.

Thank you for these comments. We appreciate the time you took to read our manuscript and share your feedback. We have added in the sample size calculation and clarified the discussion to ensure the interpretation of the results were concretely supported by the cited references.

Lastly, some crucial discussion points and limitations have not been addressed.

Major comments

P8, line 44. "This population" shall be defined more precisely.

Thank you for pointing out this omission. We have changed this to read "The prevalence of exposure to LASV in Sierra Leone is crudely estimated and largely unknown."

P11, lines 124 to 130. These are findings and conclusions and have nothing to do with introduction. Please remove from this section of the manuscript and place where appropriate.

This section has been removed from the introduction and added to the discussion.

P12, line 156. References 32 to 34 are methodological statements that have nothing to do with the content of the sentence, which is about estimated prevalence of LF in Sierra Leone. Citing references with original data that support the content of the sentence should be cited instead.

Thank you for pointing out this reference issue. We have added in references with original data that support the content of these sentences.

P15, line 227. As LASV is a very variable virus with several lineages and a limited interlineage cross-reactivity, it would be informative to state against which LASV lineage the cited performances apply (cf. ref. 47) and to discuss later what are the limitations in terms of generalizability of the results and transposability of the methods to areas where different LASV lineages circulate.

Thank you for discussing this variability within LASV. In Sierra Leone, Liberia, and Guinea, the most common lineage that is found is Lineage IV. We have added this distinction in the text and discussed the limitations in the main text, including the generalizability of results to areas where different LASV lineages circulate (i.e. Nigeria).

P23, line 395 and thereafter. Authors should be more nuanced when interpreting the results from ref. #9. A higher incidence of symptomatic LF in those older 61 is not synonymous of an increased exposure to LASV. This could reflect an increased susceptibility to develop a symptomatic disease when infected by LASV.

These sentences have been removed from the text.

P23, line 397. Same comment regarding the interpretation of the results from ref. #31. This refers to a convenience sample of hospitalized patients and does not reflect the prevalence of LASV IgG seropositivity in the source population.

These sentences have also been removed from the text.

P23, line 405. The affirmation “Mining is a known risk factor related to LASV transmission and seropositivity” is absolutely not supported by ref. #63.

The reference cited was used in error. We have corrected the text to better use the way we used the information in the provided reference. It does not directly list mining as a risk for LASV transmission, but describes how “Mining activities encourage migration to mining sites, leading to overcrowded living conditions that increase risk of infectious diseases, however it has only been anecdotally suggested as a risk factor for LASV exposure, so further investigation would be necessary.”

P24, line 415. Same remark regarding ref. #64, not supporting the affirmation in the sentence.

The reference cited here was also used in error. We have corrected the reference to support the potential of mining as a risk factor for Lassa virus infection.

Discussion and limitations section:

- Authors should mention desirability bias, as a potential limitation in the interpretation of results concerning occupational and environmental factors.
    - We have addressed the potential for desirability bias in the limitations section. “This lack of standardization could also have introduced desirability bias when interpreting the results concerning occupational and environmental factors related to LASV exposure.
- Authors should address the gap between the prevalence of LASV IgG in a population and the incidence of symptomatic LF in this population.
    - This is a great point. We have included an explanation of the limitation caused by the gap of comparison between the LASV IgG incidence vs. incidence of symptomatic LF. “There is a gap in surveillance and reporting to the MoHS in regard to LASV IgG. We can estimate the incidence of symptomatic LF in the larger population, but this doesn’t easily correlate with the seroprevalence of LASV IgG in this population.
- Authors should address the issue of non-specific cross-reactivity between LASV and other arenaviruses, some of them not being pathogenic in human. This could lead to overestimate LASV IgG prevalence in the source population.
    - We have mentioned the importance of cross-reactivity in our limitations section. “At the same time, LASV is known to experience non-specific cross-reactivity between lineages, as well as other related arenaviruses. This could lead to an over-estimation of LASV IgG prevalence in our study population.”
- Authors should discuss the possibility that seroprevalence is waning among the oldest individuals (> 45 years) as a results of :
    - Reduced environmental exposure to LASV due to behavioral changes
        - We have added in a description about the potential for changes in behavior causing lower seroprevalence.

        "This could be due to multiple reasons, potentially changes in behavior that decrease environmental exposure to LASV or more complicated immunological factors leading to immune senescence in older age."

- Immune senescence
    - This is what we hypothesize is occurring. We would love to study this more thoroughly and have included it in our discussion section. (addressed in sentence above)

Minor comments

P8, line 42. As LASV transmission may be from human to human under certain circumstances, I suggest the following modification: "Infections in human occur [mainly] after exposure…"

Thank you for this modification. We have adjusted the sentence in the manuscript.

"Infections in humans occur mainly after exposure to infected excrement or urine of the rodent-host, *Mastomys natalensis*."

Same comment P10, line 69.

We have also adjusted the sentence in the manuscript to specify the transmission from human to human.

"Lassa fever (LF), an acute viral hemorrhagic fever, is a major public health threat in West Africa. Lassa virus (LASV), the cause of LF, is transmitted to humans from the infected excrement or urine of the rodent-host, *Mastomys natalensis*."

P8, line 45. It would be more straightforward to say directly "baseline point seroprevalence of IgG antibodies to LASV in [three administrative districts of] Sierra Leone" as it is not a nationwide estimation.

Thank you for noting this. We have adjusted the sentence in the manuscript to be more direct, to avoid confusion about generalizability to Sierra Leone at the national level.

"This cross-sectional study aimed to establish a baseline point seroprevalence of IgG antibodies to LASV in three administrative districts of Sierra Leone and identify risk factors for seropositivity and LASV exposure."

P10, line 82. I suggest the following modification: "through [direct or indirect] contact…" to take into account the possibility of being infected through manipulation or consumption of foodstuff contaminated by rodent excretates.

We have specified the contact type for contaminated water or foodstuff. This will take into account the possibility of being infected by contaminated foodstuffs, not directly from rodent excreta.

"LASV is a zoonotic pathogen transmitted predominantly through direct or indirect contact with the rodent-host *Mastomys natalensis* (also known as the Natal multimammate mouse)."

P10, line 104. I suggest adding diarrhea and abdominal pain to the list of symptoms, as they are among the most frequent and can be very misleading to physicians who are not familiar with LF.

This is a great suggestion, and further illuminates the difficulties diagnosing LF. Diarrhea and abdominal pain are also misleading and can be mistaken for a large number of other infections or diseases. We have added this information to the sentence in the manuscript.

"Most signs and symptoms of LF occur 1-3 weeks after virus exposure, are highly variable, and can include fever, facial swelling, conjunctival injection, diarrhea, abdominal pain, and vomiting. Due to the non-specific clinical signs and symptoms, LF is a difficult disease to diagnose."

P12. It is quite surprising to see the Ethics approval subsection at the very beginning of the Methods section, unless it is a directive of the journal.

This is under direction from the editors of the journal. For now, we shall keep this subsection as is unless otherwise directed.

P15, line 210. I guess that seropositivity is the "dependent" variable rather than "independent".

This was a confusing way we presented the data. We have updated the manuscript and figures to reflect more clarification.

"IgG LASV seroprevalence was a proportion of IgG seropositivity measured at the household level. IgG LASV seropositivity was measured at the individual level."

P15, line 218. It would be clearer for the reader to state directly that the selected assay detects IgG direct to the LASV nucleoprotein. I suggest the following modification: "was a human anti-LASV [nucleoprotein] immunoglobulin G (IgG)…"

We have adjusted this sentence as suggested.

"The serological assay used to determine potential exposure to LASV was a human anti-LASV immunoglobulin G (IgG) antibody enzyme-linked immunosorbent assay (ELISA)."

P13, lines 161-162 and P16 lines 250-251. Redundant Fig1 caption.

We have adjusted the caption to remove the redundancy.

P17, Table 1. It would be informative to indicate the total population for each selected district.

The total population for each selected district has been presented in Figure 1.

P17, line 266. Please indicate the proportion of village within Kenema district with a prevalence >20%, as well as the total number of villages in the district (as you did for the two other districts). Same remark for chiefdoms and households thereafter.

Thank you for pointing out this omission. We have included the information related to Kenema District in the manuscript.

"Kenema District had 10 villages with greater than 20% seroprevalence, which was higher than both Port Loko and Tonkolili Districts (33.0% (n=30), Fig 2B). There were eight villages in Port Loko District (32.0% (n = 25), Fig 2C) with greater than 20% seroprevalence, which was higher than Tonkolili District, with only four villages (14.8% (n = 27), Fig 2D), though this difference was not statistically significant."
