# Supplementary material for: Seroprevalence of anti-Lassa Virus IgG antibodies in three districts of Sierra Leone: A cross-sectional, population-based study
Source: PLoS Negl Trop Dis. 2023 Feb 9;17(2):e0010938. doi: 10.1371/journal.pntd.0010938 (PMC9946222; doi:10.1371/journal.pntd.0010938)
Supplement: Attachment — Submitted filename: PNTD-D-22-00744.docx [file pntd.0010938.s003.docx]

**General comments**

This is a large sampled and well-conducted population-based study of LASV IgG seroprevalence in three district of Sierra Leone. Such a large scaled seroprevalence study have not been conducted in this part of West Africa since the 1980s. Moreover, this is the first time that improved serodiagnotics are used to conduct a large LASV seroprevalence study.

Overall, the methods used are accurate, except the calculation of sample size which is absent from the manuscript. I am less convinced by the interpretation of some of the results made by the authors. Some affirmations are not supported by the results or by the cited references.

Lastly, some crucial discussion points and limitations have not been addressed.

**Major comments**

P8, line 44. “This population” shall be defined more precisely.

P11, lines 124 to 130. These are findings and conclusions and have nothing to do with introduction. Please remove from this section of the manuscript and place where appropriate.

P12, line 156. References 32 to 34 are methodological statements that have nothing to do with the content of the sentence, which is about estimated prevalence of LF in Sierra Leone. Citing references with original data that support the content of the sentence should be cited instead.

P15, line 227. As LASV is a very variable virus with several lineages and a limited interlineage cross-reactivity, it would be informative to state against which LASV lineage the cited performances apply (cf. ref. 47) and to discuss later what are the limitations in terms of generalizability of the results and transposability of the methods to areas where different LASV lineages circulate.

P23, line 395 and thereafter. Authors should be more nuanced when interpreting the results from ref. #9. A higher incidence of symptomatic LF in those older 61 is not synonymous of an increased exposure to LASV. This could reflect an increased susceptibility to develop a symptomatic disease when infected by LASV.

P23, line 397. Same comment regarding the interpretation of the results from ref. #31. This refers to a convenience sample of hospitalized patients and does not reflect the prevalence of LASV IgG seropositivity in the source population.

P23, line 405. The affirmation “Mining is a known risk factor related to LASV transmission and seropositivity” is absolutely not supported by ref. #63.

P24, line 415. Same remark regarding ref. #64, not supporting the affirmation in the sentence.

Discussion and limitations section:

- Authors should mention desirability bias, as a potential limitation in the interpretation of results concerning occupational and environmental factors.
- Authors should address the gap between the prevalence of LASV IgG in a population and the incidence of symptomatic LF in this population.
- Authors should address the issue of non-specific cross-reactivity between LASV and other arenaviruses, some of them not being pathogenic in human. This could lead to overestimate LASV IgG prevalence in the source population.
- Authors should discuss the possibility that seroprevalence is waning among the oldest individuals (> 45 years) as a results of :
  - Reduced environmental exposure to LASV due to behavioral changes
  - Immune senescence

**Minor comments**

P8, line 42. As LASV transmission may be from human to human under certain circumstances, I suggest the following modification: “Infections in human occur [mainly] after exposure…”

Same comment P10, line 69.

P8, line 45. It would be more straightforward to say directly “baseline point seroprevalence of IgG antibodies to LASV in [three administrative districts of] Sierra Leone” as it is not a nationwide estimation.

P10, line 82. I suggest the following modification: “through [direct or indirect] contact…” to take into account the possibility of being infected through manipulation or consumption of foodstuff contaminated by rodent excretates.

P10, line 104. I suggest adding diarrhea and abdominal pain to the list of symptoms, as they are among the most frequent and can be very misleading to physicians who are not familiar with LF.

P12. It is quite surprising to see the Ethics approval subsection at the very beginning of the Methods section, unless it is a directive of the journal.

P15, line 210. I guess that seropositivity is the “dependent” variable rather than “independent”.

P15, line 218. It would be clearer for the reader to state directly that the selected assay detects IgG direct to the LASV nucleoprotein. I suggest the following modification: “was a human anti-LASV [nucleoprotein] immunoglobulin G (IgG)…”

P13, lines 161-162 and P16 lines 250-251. Redundant Fig1 caption.

P17, Table 1. It would be informative to indicate the total population for each selected district.

P17, line 266. Please indicate the proportion of village within Kenema district with a prevalence >20%, as well as the total number of villages in the district (as you did for the two other districts). Same remark for chiefdoms and households thereafter.
