# Supplementary material for: Seroprevalence of anti-Lassa Virus IgG antibodies in three districts of Sierra Leone: A cross-sectional, population-based study
Source: PLoS Negl Trop Dis. 2023 Feb 9;17(2):e0010938. doi: 10.1371/journal.pntd.0010938 (PMC9946222; doi:10.1371/journal.pntd.0010938)
Supplement: Attachment — Submitted filename: PNTD-D-22-00744_responses2_19Oct22.docx [file pntd.0010938.s005.docx]

**Overall comments from PLOST NTD Editors: (please see all of our responses in blue)**
The reviewers appreciated the attention to an important topic. Based on the reviews, we are likely to accept this manuscript for publication, providing that you modify the manuscript according to the review recommendations.

One reviewer recommends some editorial changes and clarifications that will improve the manuscript - including careful wording of Lassa fever vs. virus and some statistical method clarifications.  As editor, I find figure 5 easy enough to understand, but if you have any clarifications that you think will help the reviewer who did not like it please add them. Otherwise, you can disregard. One of the other reviewers suggests several papers you could cite in your literature review, if you wish to you can, but it is not required.

We would again like to thank the reviewers for taking additional time to read and critique our manuscript, including the additional comments and revisions we made to the original manuscript. Please find detailed responses to all critiques below.

**Reviewer's Responses to Questions**:

***Key Review Criteria Required for Acceptance?***
As you describe the new analyses required for acceptance, please consider the following:

***Methods***
-Are the objectives of the study clearly articulated with a clear testable hypothesis stated?
-Is the study design appropriate to address the stated objectives?
-Is the population clearly described and appropriate for the hypothesis being tested?
-Is the sample size sufficient to ensure adequate power to address the hypothesis being tested?
-Were correct statistical analysis used to support conclusions?
-Are there concerns about ethical or regulatory requirements being met?

Reviewer #1: (No Response)

Reviewer #2: -Are the objectives of the study clearly articulated with a clear testable hypothesis stated? YES
-Is the study design appropriate to address the stated objectives? YES
-Is the population clearly described and appropriate for the hypothesis being tested? YES
-Is the sample size sufficient to ensure adequate power to address the hypothesis being tested? UNCERTAIN
-Were correct statistical analysis used to support conclusions? YES
-Are there concerns about ethical or regulatory requirements being met? NO

Reviewer #3: (No Response)

--------------------

***Results***
-Does the analysis presented match the analysis plan?
-Are the results clearly and completely presented?
-Are the figures (Tables, Images) of sufficient quality for clarity?

Reviewer #1: (No Response)

Reviewer #2: -Does the analysis presented match the analysis plan? YES
-Are the results clearly and completely presented? NO, clarity should be improved

We have taken the time to add more clarifications to the tables and figures in the manuscript. We hope this helps clarify the results.

-Are the figures (Tables, Images) of sufficient quality for clarity? NO, maps are poorly readable

Reviewer #3: (No Response)

--------------------

***Conclusions***
-Are the conclusions supported by the data presented?
-Are the limitations of analysis clearly described?
-Do the authors discuss how these data can be helpful to advance our understanding of the topic under study?
-Is public health relevance addressed?

Reviewer #1: (No Response)

Reviewer #2: -Are the conclusions supported by the data presented? YES
-Are the limitations of analysis clearly described? YES, but some aspects still need to be discussed (see attached comments)
-Do the authors discuss how these data can be helpful to advance our understanding of the topic under study? YES
-Is public health relevance addressed? YES

Reviewer #3: (No Response)

--------------------

***Editorial and Data Presentation Modifications?***
Use this section for editorial suggestions as well as relatively minor modifications of existing data that would enhance clarity. If the only modifications needed are minor and/or editorial, you may wish to recommend “Minor Revision” or “Accept”.

Reviewer #1: (No Response)

Reviewer #2: Minor comments

P9, line 41 please correct “Lassa fever virus”=> “Lassa virus”.

Thank you for this clarification. We have corrected any mention of Lassa fever virus => Lassa virus throughout the paper.

P11, line 91. I would say “estimated” rather than “approximately” as these assumptions are based on very ancient epidemiological studies conducted in the 1980s.

This is a better representation, and the sentence has been changed to the following:

" LASV infection rates are significant across West Africa, with an estimated 100,000 to 300,000 infections and 5,000 deaths annually, which cause disruptions to social, economic and public health systems.”

P12, line 116. It is not necessary to say “antibodies” as this term is implicit in “seroprevalence”.

This is indeed a redundancy and has been removed from the sentence.

P15, line 195. Please add Fullah to the list of languages, as indicated P13, line 141.

Thank you for recognizing this omission. It has been added to the sentence:

All consents, assents, parental permissions, and questionnaires were administered orally in the resident’s preferred local dialect or language (Krio, Mende, Temne, and Fullah).

P20, line 296. Who is “that individual”?

This refers to each participant. The sentence has been changed to clarify this.

“Seropositivity is highly correlated with the district of residence for each participant.”

Reviewer #3: (No Response)

--------------------

***Summary and General Comments***
Use this section to provide overall comments, discuss strengths/weaknesses of the study, novelty, significance, general execution and scholarship. You may also include additional comments for the author, including concerns about dual publication, research ethics, or publication ethics. If requesting major revision, please articulate the new experiments that are needed.

Reviewer #1: The authors did a major revision, incorporating all my comments.
I have one last request regarding the figure 4; can you add the numbers of people per age class above each column.

Thank you for this suggestion. We have modified Figure 4 to include the numbers of people in each age class above each column on the histogram.

Reviewer #2: Major comments

P12, lines 117-118. The terms “identify correlates of prevalence for LASV acquisition” are unclear. Please clarify. Same for “potential correlates of prevalence related to LASV acquisition”, P13 line 147.

This is a bit confusing. To better clarify, we have changed this phrase to read “identify factors correlating to prevalence of LASV acquisition.” We hope this helps better describe what we are looking at in this study.

P12, line 118. Please add “nucleoprotein” between “Anti-LASV” and “IgG ELISAs” as previously requested.

We apologize for missing this previous comment. We have added this to the sentence in the manuscript to read:

“Anti-LASV nucleoprotein (NP) IgG ELISAs were used to estimate the point seroprevalence…”

P14, Table 1. There is a confusion between the prevalence of “Lassa fever”, being the acute symptomatic disease due to the infection with LASV, and the seroprevalence of “LASV IgG”. Please correct throughout the table and elsewhere in the manuscript. Also, please clarify “15 percentage points with 95% confidence”. Lastly, please clarify if “10 or 20 households” is the number of investigated households per selected villages; as it stands one could understand that it is the absolute number of investigated households.

We have updated Table 1 to be more cohesive throughout the manuscript. We are looking at the seroprevalence of Anti-LASV NP IgG, not the prevalence of Lassa fever, the symptomatic disease.

P16, line 216. “Seroprevalence/seropositivity” are the “dependent” and not the “independent” variables. Moreover, interactions terms are usually set when there is a suspicion of an interaction between two “dependent”/”explanatory” variables, and not between two “independent”/”explained” variables. Please correct and clarify.

This has been updated in the manuscript as the dependent variables, not the independent. We apologize for the mistake.

P16, line 218 and thereafter. Please indicate from the beginning of this section the detected LASV IgG epitope. I guess “nucleoprotein” in the present case. This is a key information for readers in terms of generalizability and reproducibility.

Thank you for this omission. We have clarified by adding “LASV NP epitope” as detection used.

“LASV IgG prevalence was determined from dried blood spots (DBS) by measuring the presence of IgG antibodies to the LASV NP epitope.”

Results section (in general). It is still not clear in the way the results are presented what apply to households (seroprevalence) and to individuals (seropositivity). The results should be presented in such a way as to avoid any confusion in the mind of the reader and to take up the distinction between households and individuals introduced in the methods.

We have worked through the results and tried to better clarify the difference between seroprevalence and seropositivity, including when we speak about the household level or the individual level. Each variable listed in Supplemental Table 2 also includes this distinction. We hope this helps avoid further confusion.

P19, line 268 and thereafter. The sentence “Kenema District had 10 villages with greater than 20% seroprevalence, which was higher than both Port Loko and Tonkolili Districts (33.0% (n=30), Fig 2B).” is disturbing. Please homogenize and present both absolute valued and percentage for each district rather than giving absolute values for one and percentage for the others. The same apply to the two sentences that follow.

We understand the confusion and have updated the paragraph by only including only the absolute values.

P20, lines 298 to 300. The authors state “Though not statistically significant, individuals in Tonkolili District were less likely to test positive for LASV IgG than those in Port Loko District (OR 0.812, p=.360, S4 Table).”
If not significant (and it is clearly not), it is not correct to imply that there is a difference in seropositivity between individuals living in the two districts. Please remove.
Same comment for the sentence P20, line 316-317.

These two sentences have been removed.

P23, lines 362 to 364. The authors compare their results to those of the McCormick’s study conducted in the 1980s. However, the results of both studies are not directly comparable, notably because the methods used to determine seropositivity (IFA in the McCormick’s study and ELISA detecting IgG against a specific epitope in the present study) are very different and would probably have very different diagnostic performances if applied in parallel to the samples collected here. This should be clearly explained in the discussion.

We agree that these results are not directly comparable and have added this to our discussion.

”These differences suggest a potential change in prevalence but is not directly comparable because the methods used in the McCormick et al. study was IFA rather than anti-LASV NP IgG ELISA for our study.”

Figure 5. This figure is very difficult to read and understand. Please consider another way of presenting those results.

We have added a legend giving further description to what is being depicted in Figure 5. We hope this helps clarify the figure itself.

“Fig 5. Percentage of households’ proximity to environmental factors, comparing household level LASV IgG seroprevalence (n=903)

The distance of the household to the bushes or wild land, the garbage pits or refuse pile for the household, the household’s main water source, the household’s main toilet facility, and the household’s farmland or cultivated vegetation was measured in meters by a LFOT fieldworker. Each household was categorized by the percentage of their seroprevalence. Households with more than 50% seroprevalence were categorized as high. Households with 25-50% were moderate and households less than 25% were low. The likelihood of a household having greater than 50% seroprevalence was determined in a multivariate analysis.”

Reviewer #3: The authors did address the limitations raised except for claiming that the latest studies on the epidemiology of LASV in Sierra Leone date from the 1980s, notably the studies conducted by McCormick et al. There are studies with recent data such as those cited below that I would suggest authors consider in their discussion;
O’Hearn, A.E., Voorhees, M.A., Fetterer, D.P., Wauquier, N., Coomber, M.R., Bangura, J., Fair, J.N., Gonzalez, J.-P., Schoepp, R.J., 2016. Serosurveillance of viral pathogens circulating in West Africa. Virol J 13, 163. <https://nam11.safelinks.protection.outlook.com/?url=https%3A%2F%2Fdoi.org%2F10.1186%2Fs12985-016-0621-4&amp;data=05%7C01%7Ceengel%40tulane.edu%7Cdd739a47dc574ef5dc5a08dab202781b%7C9de9818325d94b139fc34de5489c1f3b%7C0%7C0%7C638018020936202851%7CUnknown%7CTWFpbGZsb3d8eyJWIjoiMC4wLjAwMDAiLCJQIjoiV2luMzIiLCJBTiI6Ik1haWwiLCJXVCI6Mn0%3D%7C3000%7C%7C%7C&amp;sdata=PjET0PjXo8fECh0wyl%2FAL9YDUDwfNI3iChGavU0lBCg%3D&amp;reserved=0>.
Schoepp, R.J., Rossi, C.A., Khan, S.H., Goba, A., Fair, J.N., 2014. Undiagnosed acute viral febrile illnesses, Sierra Leone. Emerg Infect Dis 20, 1176–1182. <https://nam11.safelinks.protection.outlook.com/?url=https%3A%2F%2Fdoi.org%2F10.3201%2Feid2007.131265&amp;data=05%7C01%7Ceengel%40tulane.edu%7Cdd739a47dc574ef5dc5a08dab202781b%7C9de9818325d94b139fc34de5489c1f3b%7C0%7C0%7C638018020936202851%7CUnknown%7CTWFpbGZsb3d8eyJWIjoiMC4wLjAwMDAiLCJQIjoiV2luMzIiLCJBTiI6Ik1haWwiLCJXVCI6Mn0%3D%7C3000%7C%7C%7C&amp;sdata=sovo5i09ZNsfgkR2EwcfnSIVfqts5U4XB1oxB2mfBho%3D&amp;reserved=0>.

These are great suggestions to include as references in our discussion. We do appreciate the addition of these studies. Our study determined sampled from the general population, whereas these two papers suggest a sample of convenience, testing serum samples from suspected Lassa fever cases. All of these studies are presenting important seroprevalence data.
--------------------
